# Impact of Antibiotics and Proton Pump Inhibitors on Efficacy and Tolerance of Anti-PD-1 Immune Checkpoint Inhibitors

**DOI:** 10.3389/fimmu.2021.716317

**Published:** 2021-10-27

**Authors:** Quentin Giordan, Julia Salleron, Catherine Vallance, Clothilde Moriana, Christelle Clement-Duchene

**Affiliations:** ^1^ Department of Pharmacy, Centre Hospitalier Régional de Metz-Thionville, Metz, France; ^2^ Biostatistics Unit, Institut de Cancérologie de Lorraine, Vandœuvre-lès-Nancy, France; ^3^ Department of Pharmacy, Institut de Cancérologie de Lorraine, Vandœuvre-lès-Nancy, France; ^4^ Department of Internal Medicine, Centre Hospitalier Régional Universitaire de Nancy, Vandœuvre-lès-Nancy, France; ^5^ Department of Medical Oncology, Institut de Cancérologie de Lorraine, Vandœuvre-lès-Nancy, France

**Keywords:** non-small cell lung cancer (NSCLC), renal cell carcinoma (RCC), melanoma, head and neck cancer, non-small-cell lung carcinoma (NSCLC), immune checkpoint inhibitors (ICI), antibiotics, proton-pump inhibitor (PPI)

## Abstract

**Background:**

The use of antibiotics (ATB) and proton-pump inhibitors (PPI) alters the composition and diversity of the gut microbiota, which can influence the immune system, consequently interfering with response to anti-PD1 immune checkpoint inhibitors (ICI). We assessed the impact of ATB and/or PPI use on the efficacy and safety of ICI.

**Methods:**

Two hundred twelve patients treated with anti-PD1 ICI for non-small cell lung carcinoma, melanoma, upper airway & digestive tract carcinoma or renal cell carcinoma were retrospectively included. Patients having received ATB within 60 days before ICI initiation were included in the ATB+ group. Patients having received PPI within 30 days before ICI initiation were included in the PPI+ group. Four groups were thus considered: ATB-/PPI-, ATB+/PPI-, ATB-/PPI+, ATB+/PPI+. Response rate was assessed by RECIST v1.1. Overall survival (OS), progression-free survival (PFS) and adverse events, recorded using Common Terminology Criteria for Adverse Events Version 5, were compared using inverse probability of treatment weighting to account for selection bias.

**Results:**

PFS at 6 months was 56.7 %, 95%CI (49.6%; 63.2%) and 47.2 %, 95%CI (39.8%;54.1%) at 12 months. OS was 81.6%, 95%CI (75.6%; 86.2%) at 6 months, and 69.4%, 95%CI (61.9%;75.7%) at 12 months. Compared to ATB-/PPI- group, PFS was lower for the ATB+/PPI- group [Hazard ratio (HR) 1.90, 95%CI (1.41;2.57)] and the ATB-/PPI+ group [HR 1.51, 95%CI (1.11;2.05)], and lowest in the ATB+/PPI+ group [HR 3.65, 95%CI (2.75;4.84)]. For OS, the use of ATB alone or PPI alone or in combination was a risk factor for death, with each increasing HR values by a similar magnitude, and the combination of ATB and PPI did not increase risk further. AEs were observed in 78 cases (36.8%) with no significant impact of ATB or PPI use.

**Conclusions:**

This study reveals that ATB and/or PPI use can alter response to anti-PD1 ICI, and the prognosis of cancer patients. The microbiota mechanisms involved in the response to ICI should be investigated to optimize patient management.

## Introduction

Since their introduction, immune checkpoint inhibitors (ICI) have considerably improved the management of patients with solid tumors, including non-small-cell lung carcinoma (NSCLC), melanoma, renal cell carcinoma (RCC) and cancers of the upper aerodigestive tract (UADT). By stimulating and reinforcing the immune system, ICIs act by blocking two cell checkpoints that help tumors to escape immune control, namely the programmed cell death 1 (PD-1) and programmed cell-death-ligand 1 (PD-L1) (1) for pembrolizumab, nivolumab and atezolizumab, or the cytotoxic T-lymphocyte-associated protein 4 (2) for ipilimumab.

Recent systematic reviews have demonstrated the beneficial effects of ICIs on overall survival (OS) and progression-free survival (PFS) for first- (3) and second-line treatments (4) compared to chemotherapy in NSCLC patients. Long term survival has also been observed after treatment with ICIs for advanced melanoma (5) or RCC (6, 7). For patients with previously treated recurrent or metastatic UADT cancer, ICIs showed promising efficacy (8).

Recent studies have suggested that the use of antibiotics may be associated with worse outcomes in cancer patients treated with ICIs (9–12). Other studies have reported an impact of PPIs on the efficacy of ICIs (13, 14). However, the role of concomitant PPI use on the therapeutic efficacy of ICIs remains controversial, since it did not appear to be significantly associated with poorer prognosis in different cancer patients in a meta-analysis involving 1167 cancer patients from 5 studies (15). Finally, a drug-based prognostic score predicting OS, PFS and objective response rate (ORR) suggested that cumulative exposure to antibiotics and PPIs leads to progressively worse outcomes after ICI therapy (16).

The aim of our study was therefore to investigate the impact of ATB and PPI, either alone or in combination, on the efficacy and tolerance of ICI in patients with solid tumors in a real-world setting.

## Materials And Methods

### Study Approval

In accordance with current French legislation, this study was declared to the French national data protection authority (Commission Nationale Informatique & Libertés). All patients were informed that their clinical data might potentially be used retrospectively for research purposes, and they were informed of their right to refuse this usage. No refusal was recorded. The scientific and ethical committee of the Institut de Cancérologie de Lorraine, Nancy, France, approved the study.

### Data Collection

Using patients’ medical records, we retrospectively evaluated patient and tumor characteristics (gender, age, tumor location, histologic type, grade, PD-1 receptor and PD-L1 expression (percentage of tumor cells with positive membranous staining reported as the tumor proportion score), Eastern Cooperative Oncology Group Performance Status (PS), ICI use (start and end date, molecule used and treatment line, cause of treatment discontinuation), use of PPIs or ATBs (molecule and mode of administration, start and end date), appearance of immune-related AE (date, type, grade and location), PFS, OS and the best overall response to ICI.

### Patient Selection

This was a single-centre, retrospective study. All consecutive patients with NSCLC, melanoma, RCC or UADT cancer who received at least one ICI injection from 1 January 2018 and 31 December 2019 were identified from the hospital informatics database. The ICI considered were nivolumab (associated or not with ipilimumab) and pembrolizumab. Inclusion criteria were as follows: Age ≥18 years old, World Health Organization performance status ≤2, immunotherapy according to the marketing authorization or expanded access status, use of anti PD-1/PD-L1 as monotherapy or combined with anti cytotoxic T-lymphocyte-associated protein. Exclusion criteria were systemic corticosteroid therapy using ≥10 mg daily dose of prednisone within 14 days prior to ICI initiation, or use of immunosuppressive agents within 14 days prior to immunotherapy, autoimmune disease or inflammatory bowel disease (IBD), and patients previously treated by ICI or receiving concomitant chemotherapy. Patients with atezolizumab were excluded due to the number of cases being too low for analysis as well as patients with UADT cancer treated in first or third line and more, melanoma treated in third line and more and RCC treated in first or third line and more.

### Treatment and Assessment

Nivolumab was administered by intravenous infusion at a dose of 3 mg/kg every 2 weeks until August 2018 and thereafter, at a flat dose of 240mg every 2 weeks according to the recommended dosage change. In combination, patients received nivolumab at 1 mg/kg plus ipilimumab at 3 mg/kg every 3 weeks for 4 doses, followed by nivolumab at 3 mg/kg or at a flat dose of 240mg every 2 weeks. Pembrolizumab was administered at a dose of at a flat dose of 200mg every 3 weeks. Both drugs were administered until disease progression or onset of uncontrollable adverse events (AEs).

After the first ICI injection, patients were followed up every 12 weeks with medical imaging [thoraco-abdomino-pelvic computer tomography (CT) scan and brain magnetic resonance imaging (MRI)], and we recorded treatment response evaluated according to the Response Evaluation Criteria In Solid Tumors version 1.1. Prior to each ICI administration, patients were clinically assessed to identify AEs. Patients were followed up until death or data lock (31 May 2020).

The primary endpoint was progression free survival (PFS) defined as the time from immunotherapy initiation to progression or death. Efficacy was also assessed according to clinical response and OS. The best overall response rate was categorized as complete response (CR), partial response (PR), stable disease (SD) or progressive disease (PD). The ORR was based on combining CR and PR, and the disease control rate (DCR) was based on combining CR, PR and SD response. OS was defined as the time from immunotherapy initiation to death, whatever the cause. Toxicity included both non-specific and autoimmune AEs. AEs were defined according to appearance and graded according to the Common Terminology Criteria for Adverse Events, version 5. The most severe AE from ICI initiation to the end of follow-up was considered. Only grade ≥2 AEs were considered.

### ATB and PPI Exposure

Previous studies have shown that alteration of intestinal microbiota by ATB occurs within two months after ATB exposure (17, 18), while PPI treatment leads to dysbiosis after a continuous administration of 4 weeks (19). Consequently, patients who received ATB within a window of 60 days prior to ICI initiation were included in the ATB+ group, and patients who received PPIs within a window of 30 days prior to ICI initiation were included in the PPI+ group (19). Four groups were thus considered: ATB-/PPI-, ATB+/PPI-, ATB-/PPI+, ATB+/PPI+.

### Statistical Analysis

Qualitative parameters are expressed as number and percentage. Quantitative parameters are expressed as means and standard deviation or median and interquartile range based on whether or not they had a normal distribution as assessed by the Shapiro–Wilk test.

The data comparison between the 4 groups was performed using the Chi-squared test or Fisher’s exact test for qualitative variables, and the Kruskal-Wallis test or analysis of variance (ANOVA) for quantitative parameters. For all further analyses, propensity score weighting was used to adjust the results for confounding factors by using the inverse probability of treatment weighting method (20). The propensity score was estimated using multinomial logistic regression using the groups as the dependent variable and PS, gender, age, grade and the interaction between tumour location and treatment line number as independent variables. Comparison of patient characteristics after weighting on the propensity score was performed to ensure that the weighting yielded four balanced groups. The effect size of each patient characteristic was calculated with Cramer’s V statistic, with a value from 0.1 to 0.3 corresponding to a small effect size, from 0.3 to 0.5 to a moderate effect size and >0.5 to a large effect size. OS and PFS were described by the Kaplan-Meier method and compared between groups using a Cox proportional hazards model. Then, the Cox model was weighted with the propensity score, and results are expressed as hazard ratios and 95% confidence interval (95%CI).

For ORR and DCR, logistic regression was performed with the response rate as dependent variable and the group as independent variable. In a second stage, logistic regression weighted on the propensity score was performed, and results are expressed as odds ratios (ORs) and 95%CI. Statistical analyses were performed with SAS version 9.4 (SAS Institute Inc., Cary, NC, USA). The significance level was set at 5 %.

## Results

### Patient Characteristics

**Figure 1:**
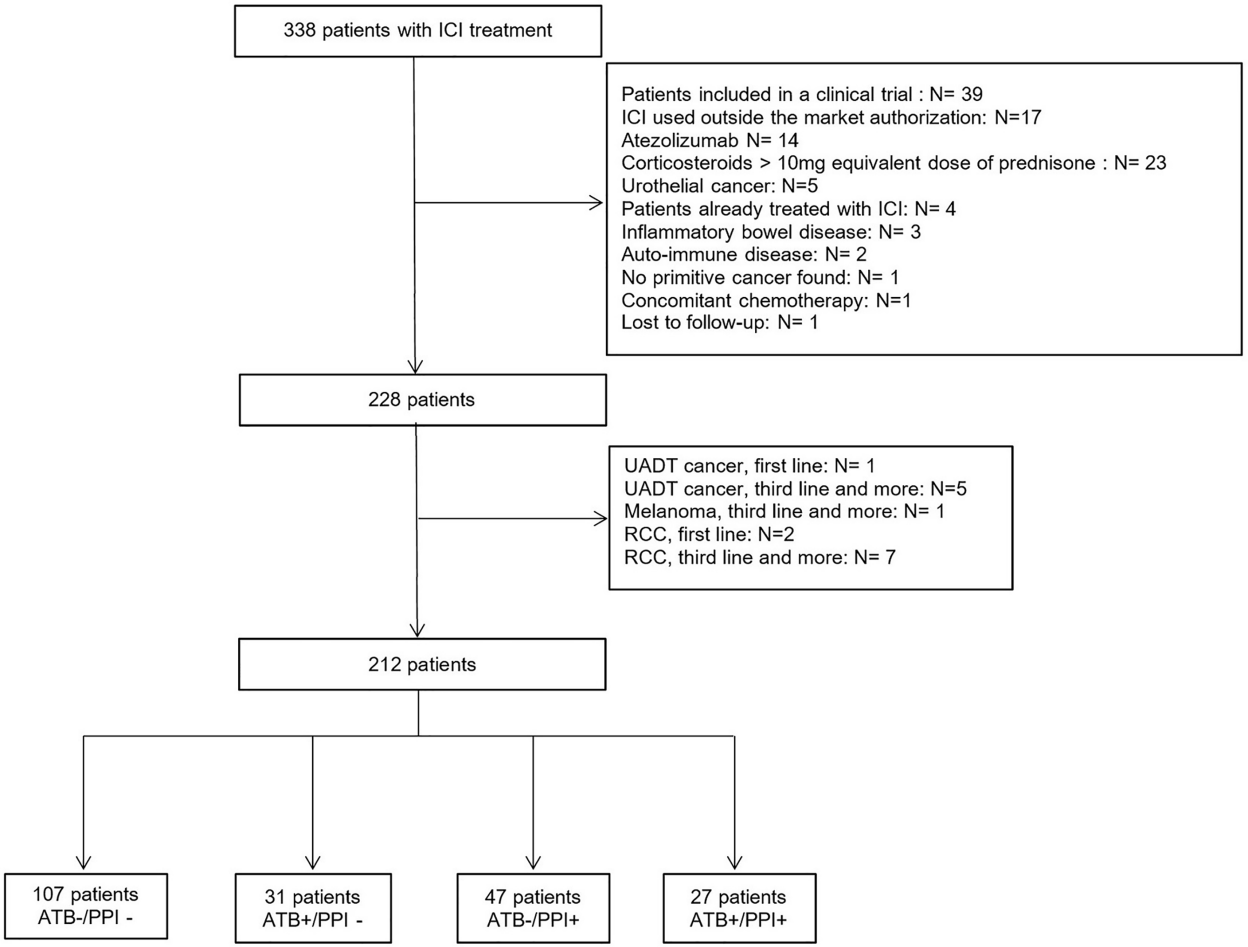
Flowchart of the study population. ATB, Antibiotics; ICI, Immune Checkpoint Inhibitors; PPI, Proton-pump inhibitors; RCC, Renal Cell Carcinoma; UADT, Upper Aero-Digestive Tract.

From 1 January 2018 to 31 December 2019, 338 patients were prescribed ICI, of whom 212 were eligible and included in this analysis (**Figure 1**).

The population was composed of 143 men (67.5 %) and 69 women (32.5%), and mean age was 63.9 ± 11.9 years. At ICI initiation, 187 patients (88.2 %) had a metastatic tumor, and 161 patients (76.3 %) had a PS ≤1. Fifty eight patients (27.2%) received ATB within 60 days prior to ICI initiation. The median duration of antibiotic therapy was 14 days [8; 98] and was ≥7 days in 54 patients (93.1%). Seventy-four patients (34.7%) were treated with PPI during the month prior to ICI initiation. The median duration of treatment was 8.4 months (4,1; 14,8). No patients had intravenous PPI administration. Considering the combination of ATB and PPI use, 107 patients (50.5 %) had neither ATB not PPI before ICI initiation (ATB-/PPI- group). While there were 31 patients (14.6 %) in the ATB+/PPI- group, 47 patients (22.2 %) in the ATB-/PPI+ group and 27 patients (12.7 %) in the ATB+/PPI+ group. As shown in **Table 1**, the demographic and baseline characteristics were balanced within groups, except for tumor location (p<0.001) and PS (p<0.001), with a Cramer’s V >0.3 corresponding to a moderate effect size. There was also a significant difference in the treatment line (p=0.002, Cramer’s V=0.26). After weighting on the propensity score, there were no longer any significant differences between groups, with all Cramer’s V close to 0.1, corresponding to a small effect size (see Additional file).

**Table 1:** Characteristics of the study population.

Characteristics		Total (N=212)	ATB-/PPI- (n=107)	ATB+/PPI- (n=31)	ATB-/PPI+ (n=47)	ATB+/PPI+ (n=27)	p-value	Effect size^2^
Age	≥ 65 years	100 (47.2)	50 (46.73)	13 (41.94)	23 (48.9)	14 (51.9)	0.89	0.05
Gender, n(%)	Men	143 (67.5)	68 (63.6)	21 (67.7)	35 (74.5)	19 (70.4)	0.59	0.09
**Tumor location, n(%)**	**Melanoma**	**76 (35.9)**	**55 (51.4)**	**3 (9.7)**	**14 (29.8)**	**4 (14.8)**	**< 0.001**	**0.31**
	**NSCLC**	**65 (30.7)**	**25 (23.4)**	**9 (29.1)**	**22 (46.8)**	**9 (33.3)**		
	**Head and Neck**	**38 (17.9)**	**9 (8.4)**	**15 (48.4)**	**2 (4.3)**	**12 (44.4)**		
	**RCC**	**33 (15.6)**	**18 (16.8)**	**4 (12.9)**	**9 (19.2)**	**2 (7.4)**		
Grade, n(%)	III	25 (11.8)	14 (13.1)	3 (9.7)	4 (8.5)	4 (14.8)	0.79	0.07
	IV	187 (88.2)	93 (86.9)	28 (90.3)	43 (91.5)	23 (85.2)		
PD-L1 expression, n(%)^1^	< 1%	13 (29.6)	5 (25.0)	2 (33.3)	4 (36.4)	2 (28.6)	NC	NC
	1 – 49%	10 (22.7)	5 (25.0)	1 (16.7)	2 (18.2)	2 (28.6)		
	≥ 50%	21 (47.7)	10 (50.0)	3 (50.0)	5 (45.5)	3 (42.9)		
**ECOG PS, n(%)**	**≤ 1**	**161 (76.3)**	**97 (91.5)**	**21 (67.7)**	**26 (55.3)**	**17 (63.0)**	**< 0.001**	**0.37**
	**≥ 2**	**50 (23.7)**	**9 (8.5)**	**10 (32.3)**	**21 (44.7)**	**10 (37.0)**		
**Treatment line, n(%)**	**1^st^**	**74 (34.9)**	**49 (45.8)**	**6 (19.4)**	**15 (31.9)**	**4 (14.8)**	**0.002**	**0.26**
	**2^nd^**	**126 (59.4)**	**54 (50.5)**	**25 (80.7)**	**28 (59.6)**	**19 (70.4)**		
	**≥ 3^rd^**	**12 (5.7)**	**4 (3.7)**	**0 (0.0)**	**4 (8.5)**	**4 (14.8)**		
Molecule used, n(%)	Nivolumab	141 (66.5)	65 (60.8)	26 (83.9)	30 (63.8)	20 (74.1)	0.085	0.17
	Pembrolizumab	71 (33.5)	42 (39.3)	5 (16.1)	17 (36.2)	7 (25.9)7)		
ICI Treatment plan, n(%)	Monotherapy	208 (98.1)	105 (98.1)	30 (96.8)	46 (97.9)	27 (100.0)	NC	NC
	Combination	4 (1.9)	2 (1.9)	1 (3.2)	1 (2.1)	0 (0.0)		

^1^Only in lungs, estimated in 46/73 patients.

^2^Cramer’s V statistic: 0.1 to 0.3 corresponds to a small effect size, from 0.3 to 0.5 to a moderate effect size and >0.5 to a large effect size. ATB, Antibiotics; PPI, Proton-pump inhibitors; NSCLC, Non-small-cell lung carcinoma; RCC, Renal Cell Carcinoma; ICI, immune checkpoint inhibitors; PD-L1, programmed cell death-ligand 1; PS, performance status; NC, not calculated.

Significant results are in bold.

The details of antibiotic and PPI use are summarized in **Tables 2**, **3**.

**Table 2:** Characteristics of antibiotic therapy for the 58 patients with ATB and according to PPI use.

Antibiotics		Total (n=58)	ATB+/PPI- (n= 31)	ATB+/PPI+ (n= 27)	p-value
Therapeutic class, n(%)	Beta lactams	32 (55.2)	17 (54.8)	15 (55.6)	0.956
	Fluoroquinolones	5 (8.6)	2 (6.5)	3 (11.1)	0.656
	Tetracyclines	18 (31.0)	11 (35.5)	7 (25.9)	0.433
	Other	9 (15.5)	3 (9.7)	6 (22.2)	0.279
Prophylactic antibiotic treatment		24 (41.4)	16 (51.6)	8 (29.6)	0.090
Indication, n(%)	Lungs	15 (45.5)	5 (35.7)	10 (52.6)	NC
	Cutaneous	12 (6.1)	1 (7.1)	1 (5.3)	
	Urinary	5 (15.1)	2 (14.3)	3 (15.8)	
	Sepsis	1 (3.0)	0	1 (5.3)	
	Other	10 (30.3)	6 (42.9)	4 (21.0)	
Mode of administration, n(%)	Oral	51 (87.9)	28 (90.3)	23 (85.2)	0.694
	Intravenous	7 (12.1)	3 (9.7)	4 (14.8)	
Duration of administration (days), n(%)	< 3	0 (0.0)	0 (0.0)	0 (0.0)	1
	3 – 6	4 (6.9)	2 (6.5)	2 (7.4)	
	≥ 7	54 (93.1)	29 (93.6)	25 (92.6)	
Treatment plan, n(%)	Monotherapy	49 (84.5)	26 (83.9)	23 (85.2)	1
	Combination	9 (15.5)	5 (16.1)	4 (14.8)	

ATB, Antibiotics; PPI, Proton-pump inhibitors; NC, not calculated.

**Table 3:** Characteristics of PPI use among the 74 patients with PPI and according to ATB use.

PPI		Total (n=74)	ATB-/PPI+ (n=47)	ATB+/PPI+ (n=27)	p-value
Therapeutic class, n (%)	Pantoprazole	31 (41.9)	19 (40.4)	12 (44.4)	0.085
	Esomeprazole	17 (23.0)	12 (25.5)	5 (18.5)	
	Lansoprazole	11 (14.9)	8 (17.0)	3 (11.1)	
	Rabeprazole	5 (6.8)	5 (10.6)	0 (0.0)	
	Omeprazole	10 (13.5)	3 (6.4)	7 (25.9)	
Corticosteroid therapy, n (%)		2 (2.7)	2 (8.3)	0 (0.0)	NC

ATB, Antibiotics; PPI, Proton-pump inhibitors; NC, not calculated.

### Effect of ATB and PPI on Response to ICI

**Figure 2:**
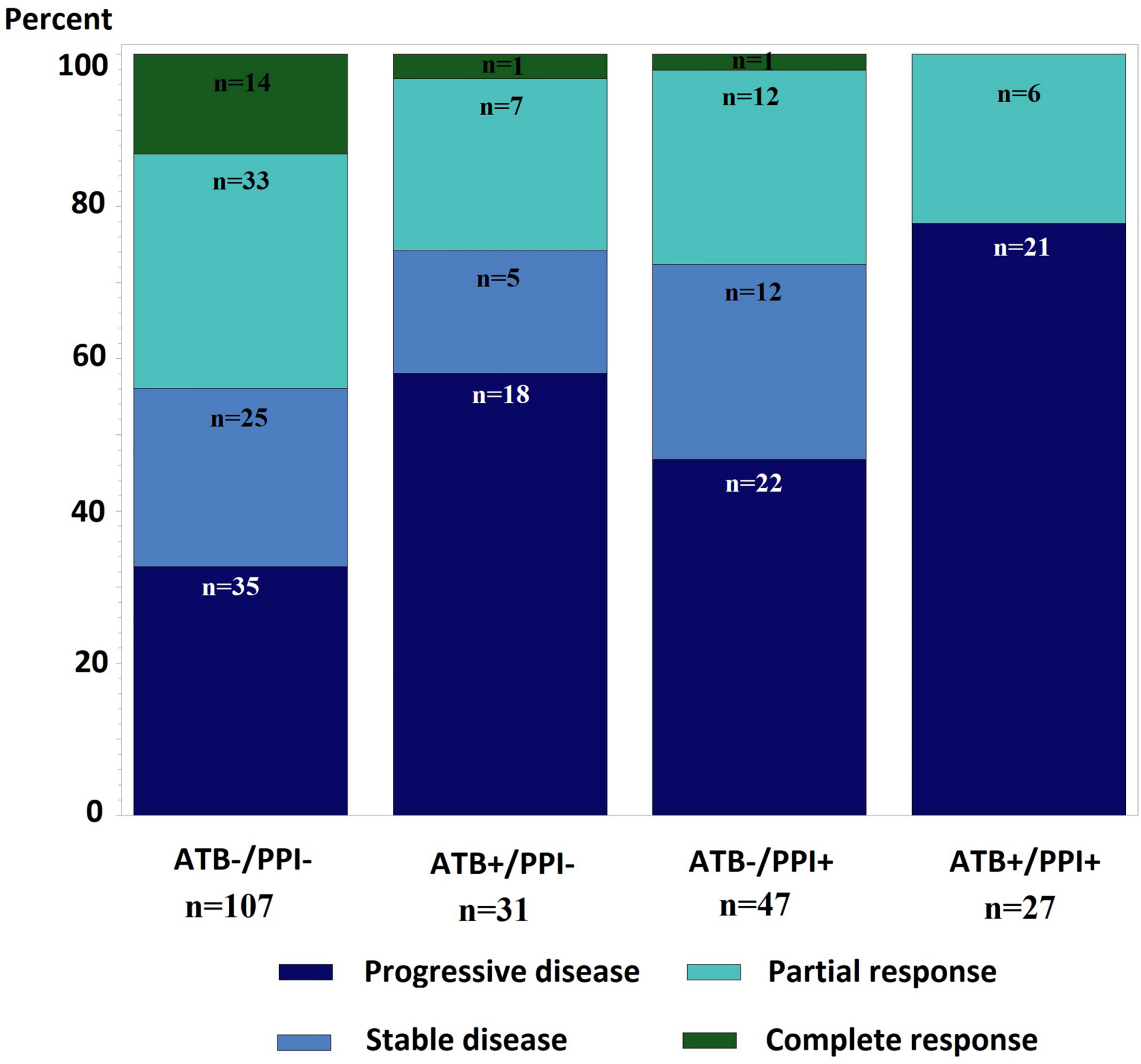
Description of overall best response according to ATB and/or PPI use.

Best overall response rates for each group are shown in **Figure 2**. The best response was achieved in 2.9 months [2.4; 5.3].

The ORR of the ATB-/PPI- group was higher than in patients treated with ATB and/or PPI (p=0.047). After weighting on propensity score, and compared to the ATB-/PPI- group, patients in the ATB-/PPI+ and ATB+/PPI+ groups had a lower probability of achieving objective response [odds ratio (OR) 0.60, 95%CI (0.40; 0.900 and OR 0.47, 95%CI (0.31; 0.72) respectively] (**Table 4**). There was no significant difference in ORR for the ATB+/PPI- compared to the ATB-/PPI- group [OR 0.81, 95%CI ([0.54; 1.22)]. Similarly, the DCR was also different according to ATB and/or PPI use (p<0.001), with a significantly lower probability of achieving disease control in the ATB+/PPI+ group (OR 0.20, 95%CI [0.13; 0.31]) compared to the ATB-/PPI- group (**Table 4**). There was no significant difference in DCR for the ATB+/PPI- [OR 0.74, 95%CI (0.49; 1.11)] and ATB-/PPI+ groups [OR 1.05, 95%CI (0.69; 1.58)]. The probability of achieving disease control was not significantly different according to prophylactic versus curative ATB (37.5% vs. 29.4%, p=0.518).

**Table 4:** Patient outcomes according to ATB and/or PPI use.

		ATB-/PPI-	ATB+/PPI-	ATB-/PPI+	ATB+/PPI+	P-value
ORR						
Univariate analysis	n(%)	47 (43.9%)	8 (25.8%)	13 (27.7%)	6 (22.2%)	0.047
	OR [95%CI]	Ref	0.44 [0.18;1.08]	0.49 [0.23;1.03]	0.36 [0.14;0.98]	
Multivariate analysis*	OR [95%CI]	Ref	0.81 [0.54;1.22]	0.60 [0.40;0.90]	0.47 [0.31;0.72]	0.003
*DCR*						
Univariate analysis	n(%)	72 (67.3%)	13 (41.9%)	25 (53.2%)	6 (22.2%)	<0.001
	OR [95%CI]	Ref	0.35 [0.15;0.80]	0.55 [0.27;1.11]	0.14 [0.05;0.37]	
Multivariate analysis*	OR [95%CI]	Ref	0.74 [0.49;1.11]	1.05 [0.69;1.58]	0.20 [0.13;0.31]	<0.001
PFS						
Univariate analysis	At 6 months	71.1%	33.0%	54.5%	30.8%	<0.001
	HR [95%CI]	Ref	3.39 [1.98;5.85]	2.23 [1.33;3.73]	4.82 [2.82;8.26]	
Multivariate analysis*	HR [95%CI]	Ref	1.90 [1.41;2.57]	1.51 [1.11;2.05]	3.65 [2.75;4.84]	<0.001
OS						
Univariate analysis	At 6 months	91.4%	66.6%	73.9%	73.1%	0.001
	HR [95%CI]	Ref	2.68 [1.22;5.92]	3.17 [1.64;6.11]	3.24 [1.47;7.14]	
Multivariate analysis*	HR [95%CI]	Ref	2.11 [1.37;3.26]	1.89 [1.23;2.90]	2.12 [1.37;3.27]	0.002

*After weighting on the propensity score. ATB, Antibiotics; PPI, Proton-pump inhibitors; ORR, objective response rate; DCR, disease control rate; PFS, Progression Free Survival; OS, Overall Survival; OR, Odds ratio; HR, Hazard ratio; CI, Confidence interval; Ref, Reference.

### Effect of ATB and PPI Use on PFS and OS

**Figure 3:**
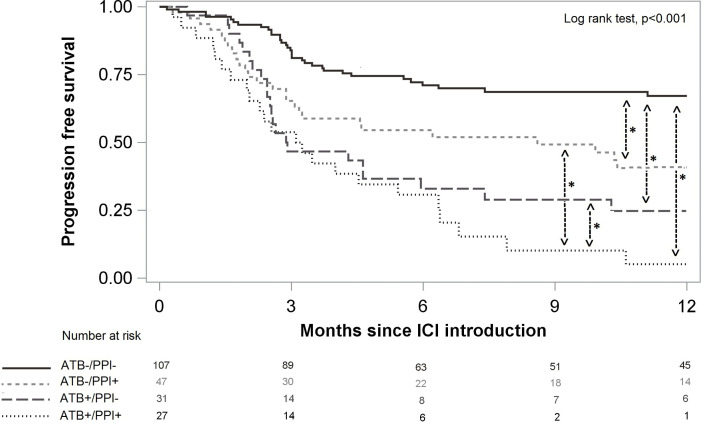
Progression free survival according to ATB and/or PPI use. Significant differences after multivariate analysis are symbolized with a star.

Median follow-up time was 10 months [interquartile range from 5 to 12 months). PFS at 6 months was 56.7 %, 95%CI (49.6%; 63.2%) and 47.2 %, 95%CI (39.8%; 54.1%)] at 12 months. Patients not treated with either ATB or PPI had better PFS than patients treated with ATB and/or PPI (p<0.001, **Figure 3**). After weighting on the propensity score, and compared to the ATB-/PPI- group, PFS was worse in the ATB+/PPI- [Hazard ratio (HR) 1.90, 95%CI (1.41; 2.57)] and ATB-/PPI+ groups [HR 1.51, 95%CI (1.11; 2.05)], and even more unfavourable in the ATB+/PPI+ group (HR 3.65, 95%CI [2.75; 4.84]).

**Figure 4:**
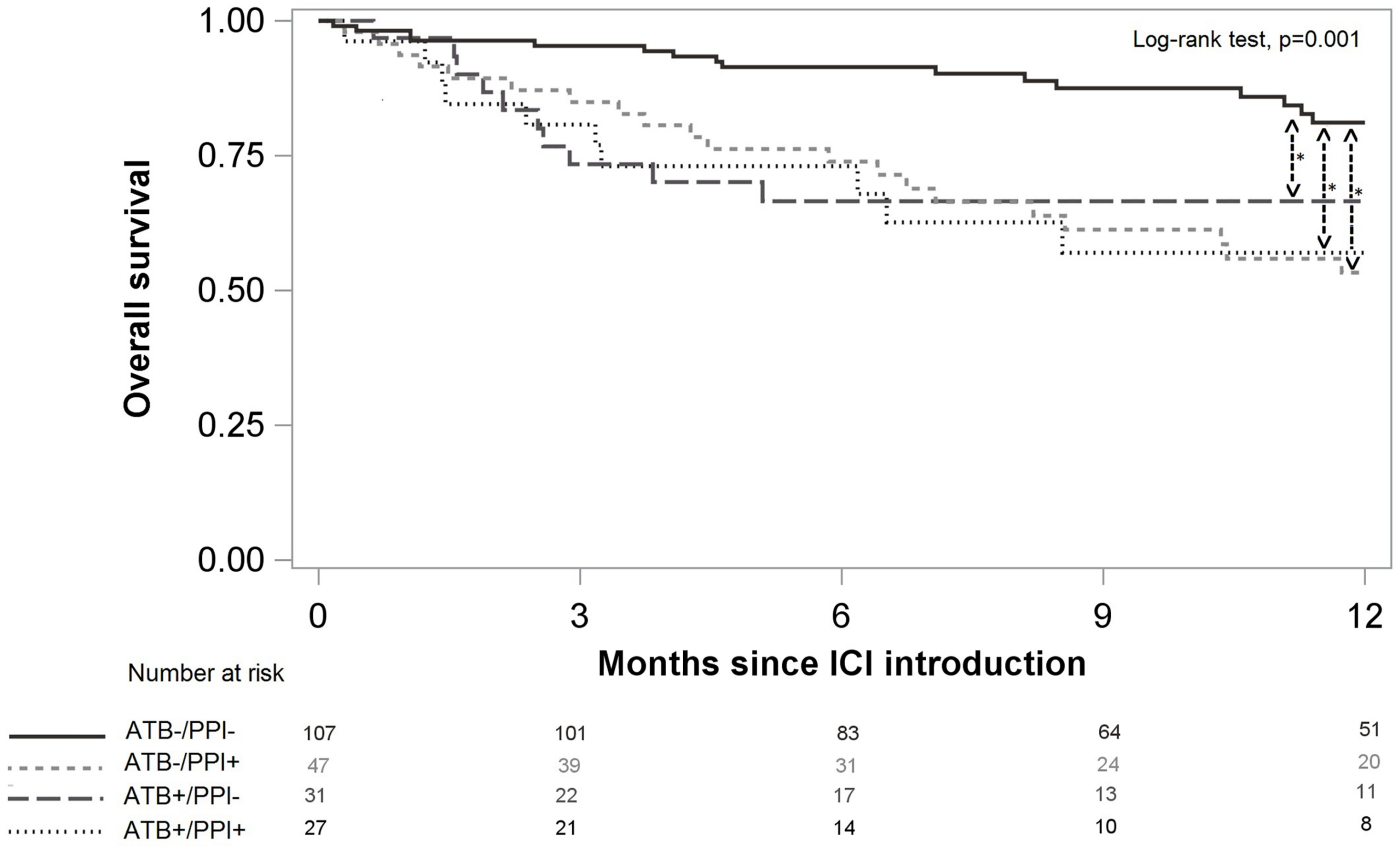
Overall survival according to ATB and/or PPI use. Significant differences after multivariate analysis are symbolized with a star.

OS was 81.6%, 95%CI (75.6%; 86.2%) at 6 months and 69.4%, 95%CI (61.9%; 75.7%) at 12 months. Patients not treated with either ATB or PPI had better OS than patients treated with ATB and/or PPI (p=0.001, **Figure 4**). After weighting on the propensity score, use of ATB alone, use of PPI alone, and the combination of both, were significant risk factors for death, and increased the risk by a similar magnitude (**Table 4**). There was no significant difference according to prophylactic versus curative ATB on PFS [HR 0.77, 95%CI (0.42;1.38)] nor on OS [HR 0.67, 95%CI (0.27;1.69)].

### Effect of ATB and PPI on AEs

Among the 212 patients, AEs were observed in 78 patients (36.8 %). The median time to appearance of non-specific AEs was 28 days (15; 87) versus 84 days (42; 147) days for auto-immune AEs. No significant difference in the rate of AEs was observed between groups (p=0.58). The AEs are detailed by group in **Table 5**.

**Table 5:** Adverse events in the whole population and in each group.

	Total (n=212)	ATB-/PPI- (n=107)	ATB+/PPI- (n=31)	ATB-/PPI+ (n=47)	ATB+/PPI+ (n=27)	p-value
Adverse events, n(%)	78 (36.8)	43 (40.2)	10 (32.3)	14 (29.8)	11 (40.8)	0.577
≥1 Non-specific events, n(%)	59 (27.8)	30 (28.0)	9 (29.0)	10 (21.3)	10 (37.0)	0.538
Grade, n(%)						
2	50 (23.6)	27 (25.2)	6 (19.4)	8 (17.0)	9 (33.3)	
3	9 (4.3)	3 (2.8)	3 (9.7)	2 (4.3)	1 (3.7)	
Asthenia, n(%)						
2	35 (16.5)	15 (14.0)	4 (12.9)	7 (14.9)	9 (33.3)	
3	7 (3.3)	2 (1.9)	3 (9.7)	1 (2.1)	1 (3.7)	
Cutaneous eruption, n(%)						
2	16 (7.6)	12 (11.2)	2 (6.5)	2 (4.3)	0 (0.0)	
3	0 (0.0)	0 (0.0)	0 (0.0)	0 (0.0)	0 (0.0)	
Diarrhoea, n(%)						
2	9 (4.3)	6 (5.6)	2 (6.5)	0 (0.0)	1 (3.7)	
3	2 (0.9)	1 (0.9)	0 (0.0)	1 (2.1)	0 (0.0)	
≥1 Auto-immune events, %(n)	40 (18.9)	26 (24.3)	4 (12.9)	7 (14.9)	3 (11.1)	0.228
Grade, n(%)						
2	34 (16.0)	21 (19.6)	3 (9.7)	7 (14.9)	3 (11.1)	
3	6 (2.8)	5 (4.7)	1 (3.2)	0 (0.0)	0 (0.0)	
Appearance, n(%)						
< 8 weeks	14 (35.0)	10 (38.5)	1 (25.0)	2 (28.6)	1 (33.3)	
≥ 8 weeks	26 (65.0)	16 (61.5)	3 (75.0)	5 (71.4)	2 (66.7)	
Liver, n(%)						
2	0 (0.0)	0 (0.0)	0 (0.0)	0 (0.0)	0 (0.0)	
3	4 (1.9)	3 (2.8)	1 (3.2)	0 (0.0)	0 (0.0)	
Lung, n(%)						
2	4 (1.9)	0 (0.0)	1 (3.2)	3 (6.4)	0 (0.0)	
3	0 (0.0)	0 (0.0)	0 (0.0)	0 (0.0)	0 (0.0)	
Thyroid, n(%)						
2	16 (7.6)	11 (10.3)	2 (6.5)	2 (4.3)	1 (3.7)	
3	0 (0.0)	0 (0.0)	0 (0.0)	0 (0.0)	0 (0.0)	
Adrenal glands, n(%)						
2	11 (5.2)	9 (8.4)	1 (3.2)	1 (2.1)	0 (0.0)	
3	0 (0.0)	0 (0.0)	0 (0.0)	0 (0.0)	0 (0.0)	
Colon, n(%)						
2	6 (2.8)	4 (3.7)	0 (0.0)	1 (2.1)	1 (3.7)	
3	1 (0.5)	1 (0.9)	0 (0.0)	0 (0.0)	0 (0.0)	
Other, n(%)						
2	1 (0.5)	0 (0.0)	0 (0.0)	0 (0.0)	1 (3.7)	
3	1 (0.5)	1 (0.9)	0 (0.0)	0 (0.0)	0 (0.0)	

ATB, Antibiotics; PPI, Proton-pump inhibitors.

## Discussion

This study shows that PFS in patients not treated with either antibiotics or PPI prior to initiation of ICI treatment is superior to that of patients treated with ATB and/or PPI before ICIs. Moreover, the concomitant administration of ATB and PPI prior to ICI initiation may possibly be associated with a greater magnitude of negative effect on PFS. Regarding OS, ATB and PPI both exert a negative impact on OS, albeit without signs that the combination has an effect of increased magnitude. No significant impact of ATB or PPI use on ICI toxicity was observed.

Our findings are consistent with previous reports (9–12, 21, 22) indicating that the use of ATB is associated with poorer outcome and may influence the efficacy of ICI. As in recent studies (13, 14), we also found in multivariate analyses that PPI use increased the risk of progression and death, albeit less than twofold. Data on the concomitant use of PPI and ATB are sparse (14, 16) but are in line with our results. In our study, we show that the combination of ATB plus PPI, compared to either ATB or PPI alone, had a negative impact on PFS but not on OS. The significant results for PFS and not for OS could be explained by the fact that post-treatments can affect OS but also by a too short median follow-up time to assess the impact on OS.

The originality of our study in this regard is the inclusion of 4 groups of patients according to the use of PPI and/or ATB, and consequently, their synergistic effect, instead of considering PPI use as a patient characteristic (23). Chalabi at al (14) studied patients treated with atezolizumab, and showed that PPI use was associated with a greater risk of progression or death, while ATB use was associated with a greater risk of death. They also concluded that an effect of the concomitant use of ATB plus PPI could not be ruled out, since a deleterious effect of the combination was demonstrated in the atezolizumab group. Buti at al (16). developed a drug-based prognostic score ranging from 0 (best prognosis, the patient did not take any of corticosteroids, systemic antibiotics or PPIs) to 4 (worst prognosis, the patient was on treatment with corticosteroids, systemic antibiotics and PPIs at ICI initiation). They assigned 1 point for PPIs and 1 point for ATB, suggesting an additive effect, and demonstrated that the higher the score, the worse the PFS and OS. More recently, Cortellini et al. (24) showed that certain co-medications, including ATB and PPIs, had a negative impact on the efficacy of ICI in stage IV cancer patients. However, in this study, only prophylactic ATB decreased PFS and OS. In our study, the impact of prophylactic and curative ATB on efficacy was not significantly different and the therapeutic class of ATB could not be assessed due to the small sample size for each.

The reduced efficacy of ICI after ATB use may be related to gut dysbiosis. The administration of ATB could alter host microbiota symbiosis, with a decrease in the immunostimulatory function of the microbiota, leading to reduced efficacy of the ICI. Some commensal bacteria (*Bifidobacterium*) help antitumor immunity to develop, and enhance the intratumoral infiltration of dendritic cells and TH1 cells (25). The same was shown with *Faecalibacterium*, reported to increase PFS *via* the development of CD8+ cells in the tumor microenvironment (26). The abundance and diversity of the gut microbiota are also altered by the use of PPI. Various hypotheses can be put forward to explain this phenomenon. Firstly, the use of PPI may affect the abundance and diversity of *Bifidobacterium* and *Ruminococcaceae*, both indispensable to immunity (27, 28). Secondly, the use of PPI can affect immunity increasing the pH of the tumor microenvironment. In preclinical models, PPIs inhibit the vacuolar H+ATPase pump and reverse the pH gradient of an acidic TAM (29). In addition, the activation of PPIs would promote the recruitment of M2 subtype macrophages as well as the production of pro-inflammatory cytokines (interleukin 1, interleukin 7, nitric oxide, tumor necrosis factor alpha) (30). In this way, the immunosuppressive activity of the tumor microenvironment would be lost, and the activity of the ICI would be decreased.

Regarding ICI tolerance, no significant difference was observed for either non-specific (p=0.54) or autoimmune AEs (p=0.23), regardless of their grade. Our results are consistent with other studies (31, 32). In a preliminary study among patients with immune-related colitis treated with ICI prior to fecal microbiota transplantation (33), it was shown that fecal microbiota transplantation might alter the LyTCD8+/LyTreg immune balance, to the benefit of Treg Foxp3+ cell infiltration in the intestinal mucosa. Thus, an immunosuppressant and anti-inflammatory gut environment might help to control and reduce immune-related colitis. The *Bacteroidetes* phylum could play an immunomodulating role, enhancing the differentiation of naïve T cells into Treg cells, thus reducing colitis (34). It could thus be associated with improved autoimmune tolerance but decreased efficacy of ICIs. A prospective study of 26 patients with metastatic melanoma showed that the microbiota of responders to ICI was enriched with *Firmicutes*, in particular *Faecalibacterium prausnitzii* and *Gemmiger formicilis*. The butyrate-producing bacteria seem to decrease the efficacy of ICI and induced poorer tolerance to ICI due to the development of immune-related colitis (35).

This study has some limitations. Firstly, it was a retrospective and single-centre study. Moreover, as few patients presented AE, studies including larger sample sizes are needed to determine the impact of ATB and/or PPI use on the occurrence of AEs. Certain parameters likely to modulate the diversity and composition of the microbiota were not recorded, such as diet or use of other drugs that could alter the microbiota. A high-fat diet rich in animal proteins increases biliary secretion and produces overexpression of the *Bacteroides* gene in order to convert these acids into short-chain fatty acids (36). Some treatments that were not considered in our study might also affect the microbiota, and could potentially interfere with immunity, such as second-generation antipsychotics (clozapine, olanzapine and risperidone), which are thought to cause dysbiosis, decreasing the abundance of *A. muciniphila* in the microbiota of non-obese patients (37), as well as non-steroidal anti-inflammatory drugs, which are thought to modulate interindividual diversity in the gut microbiome (16, 38).

The use of ATB and/or PPI affects response to ICIs and prognosis of patients with cancer. This study shows the impact of combined ATB/PPI therapy, which is often administered in cancer patients. However, further prospective studies with larger populations are essential to confirm these findings. Faecal analysis would also help to identify the commensal bacterial species that mediate patient prognosis. The modulation of microbiota could count among new therapeutic targets to ensure optimal management of patients treated with ICI.

## Data Availability Statement

The raw data supporting the conclusions of this article will be made available by the authors, without undue reservation.

## Ethics Statement

Ethical review and approval was not required for the study on human participants in accordance with the local legislation and institutional requirements. The ethics committee waived the requirement of written informed consent for participation.

## Author Contributions

CV and CC-D designed and directed the project. QG and CM interpreted the patient data. JS performed statistical analyses. QG and JS wrote the manuscript. All authors contributed to the article and approved the submitted version.

## Conflict of Interest

The authors declare that the research was conducted in the absence of any commercial or financial relationships that could be construed as a potential conflict of interest.

## Publisher’s Note

All claims expressed in this article are solely those of the authors and do not necessarily represent those of their affiliated organizations, or those of the publisher, the editors and the reviewers. Any product that may be evaluated in this article, or claim that may be made by its manufacturer, is not guaranteed or endorsed by the publisher.
