# Supplementary material for: Impact of Antibiotics and Proton Pump Inhibitors on Efficacy and Tolerance of Anti-PD-1 Immune Checkpoint Inhibitors
Source: Front Immunol. 2021 Oct 27;12:716317. doi: 10.3389/fimmu.2021.716317 (PMC8578856; doi:10.3389/fimmu.2021.716317)
Supplement: Supplementary file 1 [file Table_1.docx]

Supplementary Material

**Supplementary file 1** : Comparisons of patients characteristics after weighting on the propensity score

| Characteristics | | ATB-/PPI-  (n=107) | ATB+/PPI-  (n=31) | ATB-/PPI+  (n=47) | ATB+/PPI+  (n=27) | p-value | Effect size ^∆^ |
| --- | --- | --- | --- | --- | --- | --- | --- |
| Age | ≥ 65 years | 51.8% | 43.4% | 43.8% | 64.6% | <0.001 | 0.17 |
| Gender | Men | 65.2% | 74.8% | 67.7% | 73.8% | 0.109 | 0.09 |
|  | Women | 34.8% | 25.2% | 32.3% | 26.2% |  |  |
| Tumor location | Melanoma | 35.8% | 31.9% | 37.1% | 29.6% | 0.126 | 0.08 |
|  | NSCLC | 30.0% | 28.1% | 32.8% | 33.2% |  |  |
|  | Head and Neck | 16.9% | 21.6% | 10.0% | 21.5% |  |  |
|  | RCC | 17.2% | 18.4% | 20.1% | 15.8% |  |  |
| Grade | III | 9.6% | 4.8% | 5.9% | 12.4% | 0.029 | 0.11 |
|  | IV | 90.4% | 95.2% | 94.1% | 87.6% |  |  |
| ECOG PS | ≤ 1 | 77.8% | 80.9% | 71.9% | 71.1% | 0.079 | 0.09 |
|  | ≥ 2 | 22.2% | 19.1% | 28.1% | 28.9% |  |  |
| Treatment line, | 1 | 35.2% | 39.0% | 35.4% | 31.3% | 0.489 | 0.06 |
|  | ≥2 | 64.8% | 61.0% | 64.6% | 68.7% |  |  |
| Molecule used | Nivolumab* | 65.5% | 71.2% | 64.9% | 55.0% | 0.011 | 0.12 |
|  | Pembrolizumab | 34.5% | 28.2% | 35.1% | 45.0% |  |  |

Results expressed as weighting percentage with each group

^∆^ Cramer’s V statistics from 0.1 to 0.3 corresponding to a small effect size, from 0.3 to 0.5 to a moderate effect size and >0.5 to a large effect size.

*4 patients in combination with ipilimumab

Abbreviations : *ATB* Antibiotics *PPI* Proton-pump inhibitors *NSCLC* Non-small-cell lung carcinoma, *RCC* Renal Cell Carcinoma *PS* performance status
